# A High-Accuracy Star Centroid Extraction Method Based on Kalman Filter for Multi-Exposure Imaging Star Sensors

**DOI:** 10.3390/s23187823

**Published:** 2023-09-12

**Authors:** Wenbo Yu, Hui Qu, Yong Zhang

**Affiliations:** 1 College of Mechanical and Electrical Engineering, Inner Mongolia Agricultural University, Hohhot 010018, China; hityuwenbo@imau.edu.cn; 2 Peng Cheng Laboratory, Department of Mathematics and Theory, Shenzhen 518055, China; zhangy21@pcl.ac.cn

**Keywords:** multi-exposure imaging, star sensor, star centroid extraction, Kalman Filter, star point prediction window, high attitude update rate

## Abstract

A multi-exposure imaging approach proposed in earlier studies is used to increase star sensors’ attitude update rate by *N* times. Unfortunately, serious noises are also introduced in the star image due to multiple exposures. Therefore, a star centroid extraction method based on Kalman Filter is proposed in this paper. Firstly, star point prediction windows are generated based on centroids’ kinematic model. Secondly, the classic centroid method is used to calculate the coarse centroids of the star points within the prediction windows. Lastly, the coarse centroids are, respectively, processed by each Kalman Filter to filter image noises, and thus fine centroids are obtained. Simulations are conducted to verify the Kalman-Filter-based estimation model. Under noises with zero mean and ±0.4, ±1.0, and ±2.5 pixel maximum deviations, the coordinate errors after filtering are reduced to about 37.5%, 26.3%, and 20.7% of the original ones, respectively. In addition, experiments are conducted to verify the star point prediction windows. Among 100 star images, the average proportion of the number of effective star point objects obtained by the star point prediction windows in the total object number of each star image is calculated as only 0.95%. Both the simulated and experimental results demonstrate the feasibility and effectiveness of the proposed method.

## 1. Introduction

The star sensor is currently known as the most accurate attitude measurement instrument, and it also has the characteristics of no drift and autonomy [1,2,3,4,5,6,7,8,9,10,11]. Therefore, the star sensor has been widely used in the field of aerospace navigation [12,13,14,15,16,17,18,19,20,21,22]. However, the attitude update rate (AUR) of the traditional star sensor is usually 4–10 Hz, which is too low to accomplish navigation tasks independently. In order to improve the AUR, an integrated navigation system of a star sensor and inertial measurement unit (IMU) has become a kind of classic solution [23,24,25,26,27,28,29]. The attitude data of the star sensor have no drift but the update rate is low, while the update rate of the IMU is high, but the attitude data drift with time. When the above two navigation devices are combined, the attitude data of the star sensor can be regularly used to correct the data drift of the IMU. The integrated navigation system can output the attitude data without drift and with a high update rate. Additionally, when a three-field-of-view star sensor is adopted [30], the AUR can reach to three times of that of a single-field-of-view star sensor. This is because the three fields can be used to expose and output the attitude data alternately. Nevertheless, both the above two schemes also have some obvious disadvantages, such as a large volume, heavy weight, high power consumption, and so on, which are not conducive to the overall development of an attitude measurement and control system towards light and miniaturization. 

In contrast, research on the improvement method for the AUR of a single star sensor seems to have more extensive application value. Zhong et al. [31] and Mao et al. [32], respectively, studied the working characteristics of the star sensor and then divided its working process into three stages, i.e., star image exposure imaging and star image pixel data transmission and processing, as well as star tracking and attitude calculation. Subsequently, they proposed a parallel pipeline processing method, and thus the above three working stages could be pipelined, so as to improve the AUR of the star sensor. In this situation, the AUR is totally determined by the most time-consuming one of the above three working stages. For the conventional star sensor, a long exposure time must be conducted to obtain a sufficient star sensitivity due to the low sensitivity of the image detector. Therefore, the exposure time is the main bottleneck of the AUR of the conventional star sensor. In order to reduce the exposure time, some scholars have introduced high-sensitivity image detectors into the field of star sensors, such as electron-multiplying charge-coupled devices (EMCCD) and intensified charge-coupled devices (ICCD), etc. The shortening of the exposure time improves the AUR of star sensors to a certain extent. At this time, the transmission and processing times of the star image pixel data become a new bottleneck of the AUR. To further improve the AUR, Wang et al. [33] proposed a star centroid extraction method based on distributed parallel super blocks, and Ding et al. [34] also proposed a multichannel star centroid extraction method. The above two methods both utilized the idea of parallel processing to extract the star centroid, thus effectively reducing the pixel data processing time. Nevertheless, the transmission time of pixel data could still not be significantly reduced because of the restriction in the output capacity of the image detector itself. Meanwhile, Liebe’s research [35] pointed out that the attitude measurement accuracy of the star sensor is correspondingly improved with an improvement in the pixel resolution of the image detector, without changing other parameters. Therefore, large-array image detectors have been gradually applied to the field of star sensors. In this case, the transmission time consumption of star image pixel data is more serious, which greatly limits the AUR’s further improvement. In order to break the above limitation, some scholars have proposed a rolling shutter exposure imaging method [36,37,38,39]. In this method, each star point in a single star image is actually the imaging result at different moments. When the attitude tracking and single star point measurement are combined, a set of attitude data can be obtained by each star point, thus resulting in the AUR’s improvement. Unfortunately, the attitude measurement’s accuracy is seriously affected by the random error of the single star centroid, which makes it difficult to ensure the attitude measurement accuracy of star sensors. 

In order to significantly improve the AUR, as well as overcome the shortcomings of the above methods, a multi-exposure imaging approach was proposed in the authors’ previous research [40,41]. *N* times of short exposure can be adaptively inserted into one normal exposure period according to the angular velocity of the carrier, so that the star spots at *N* moments can be recorded in a single star image. When the star tracking and attitude calculation are carried out according to *N* moments, respectively, the attitude information corresponding to *N* moments can be obtained, which is equivalent to increasing the AUR by *N* times. However, due to multiple exposures, serious noises and false objects are also introduced in the star image, which is not conducive to practical applications. For this reason, this paper proposes a star centroid extraction method based on Kalman Filter. It has a significant effect on removing false objects and improving the centroid accuracy, thus benefiting the practical application of the multi-exposure imaging star sensor (MEISS). 

The remainder of this paper is organized as follows. Section 2 details the principle of the high-accuracy star centroid extraction method, including the star point prediction windows, centroiding, and Kalman Filter. Both simulations and experiments in Section 3 are conducted to demonstrate the feasibility and effectiveness of the proposed method. Section 4 describes the discussion of the simulated and experimental results. Finally, conclusions are drawn in Section 5. 

## 2. Principle of High Accuracy Star Centroid Extraction Method

**Figure 1:**
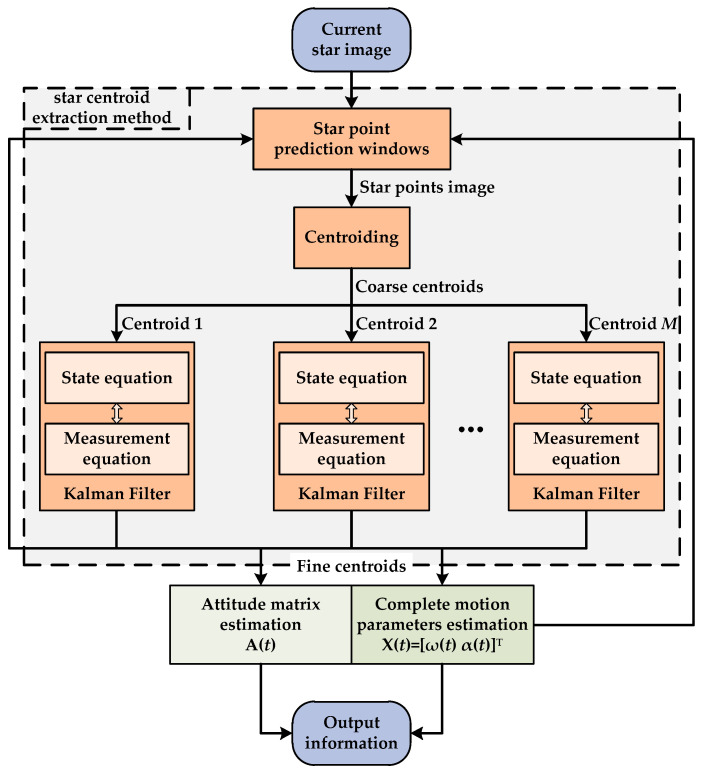
Flow chart of the proposed star centroid extraction method.

As described in the authors’ previous research [40], the multi-exposure imaging approach can be adopted to improve the star sensor’s AUR by *N* times. Nevertheless, due to the multiple exposures of an image detector, more severe imaging noises, as well as more fake star points, will be inevitably introduced into the whole star image. This is unfavorable for the accurate centroid extraction of true star points, thus affecting the MEISS’s accuracy. To ensure the MEISS’s effective application, this study proposes a high-accuracy star centroid extraction method based on Kalman Filter, which is illustrated in Figure 1. 

As shown in Figure 1, the proposed star centroid extraction method can be divided into three parts, i.e., star point prediction windows, centroiding, and Kalman Filter. Firstly, the star point prediction windows are generated by combining the fine centroids of the star spots in the former star image, as well as the complete motion parameters **X**(*t*) (including the angular velocity **ω**(*t*) and angular acceleration **α**(*t*)) [41]. The current star image will be processed by the star point prediction windows and then only the star points within the prediction windows can be transmitted to the next step. Secondly, the classic centroid method is used to calculate the centroids of the above star points. These centroids inevitably include many image noises and thus are just coarse centroids. Lastly, the coarse centroids are, respectively, processed by each Kalman Filter to filter the image noises and thus fine centroids are obtained. By using the fine centroids, both the attitude matrix and complete motion parameters can be estimated as the final output information. Meanwhile, the current fine centroids and complete motion parameters can be utilized to generate the next star point prediction windows. Therefore, the high-accuracy star centroid extraction can be constantly realized in such circulation. 

### 2.1. Star Point Prediction Windows

**Figure 2:**
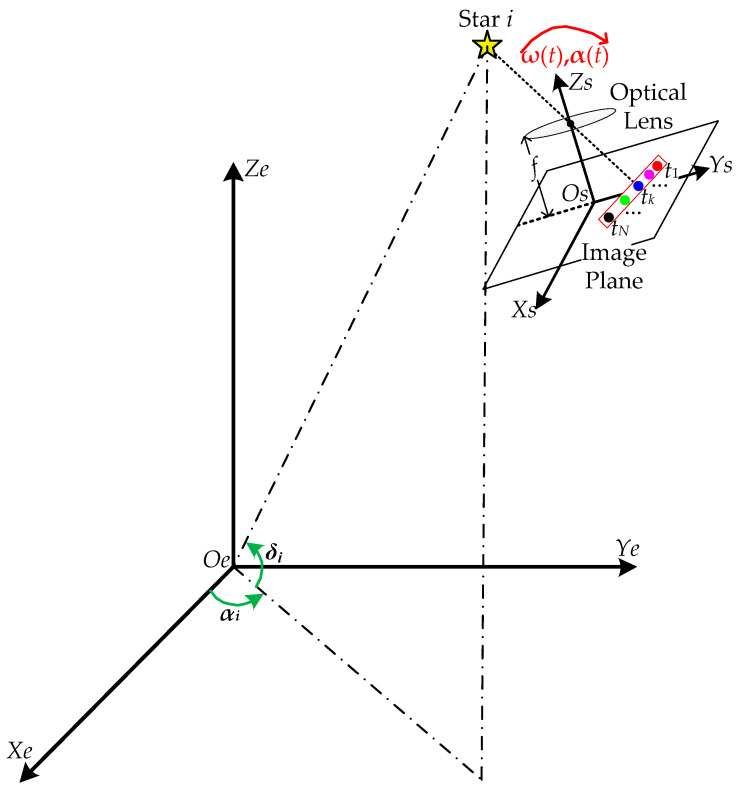
Schematic diagram of the MEISS’s basic working principle.

Figure 2 shows the MEISS’s basic working principle. *Os*-*XsYsZs* is the coordinate frame of the star sensor. Its coordinate origin *Os* is located at the center of the image plane of the image detector. The *Xs-Os-Ys* plane coincides with the image plane, and the *Xs*-axis and *Ys*-axis are, respectively, parallel to the horizontal and vertical directions of the image plane. The *Zs*-axis points to the direction of the bore sight. *Oe*-*XeYeZe* is the celestial coordinate frame. It is an inertial frame and its coordinate origin *Oe* is located at the center of the earth. The *Xe-Oe-Ye* plane coincides with the celestial equator plane. The *Xe*-axis points to the vernal equinox and the *Ze*-axis points to the celestial north pole. The *Ye*-axis is determined by the right-handed coordinate frame. 

There are a large number of stars who are moving quite slowly with time in the whole celestial sphere. After the correction of precession and nutation [42], the positions of the stars can be considered as time-independent constants. Taking Star *i* as an example, its right ascension (the rotation angle from the *Xe*-axis to the projection of Star *i* on the *Xe-Oe-Ye* plane) and declination (the rotation angle from the projection of Star *i* on the *Xe-Oe-Ye* plane to Star *i*) are, respectively, (*α_i_*, *δ_i_*), and then its reference unit vector in the celestial coordinate frame *Oe*-*XeYeZe* can be expressed as:

(1) $$V_{i}=\left[\begin{array}{c} \cos\delta_{i}\cos\alpha_{i}\\ \cos\delta_{i}\sin\alpha_{i}\\ \sin\delta_{i} \end{array}\right].$$

When Star *i* is imaged by the MEISS, there will be a total of *N* imaging star spots of Star *i* in one star image due to the exposure times *N*. Taking the imaging time *t_k_* (*k* = 1,2,…,*N*) as an example, the observation unit vector of Star *i* in the MEISS’s coordinate frame *Os*-*XsYsZs* can be expressed as:

(2) $$W_{i}(t_{k})=\frac{1}{\sqrt{x_{i}{\left(t_{k}\right)}^{2}+y_{i}{\left(t_{k}\right)}^{2}+f^{2}}}\left[\begin{array}{c} -x_{i}(t_{k})\\ -y_{i}(t_{k})\\ f \end{array}\right],$$

where (*x_i_*(*t_k_*), *y_i_*(*t_k_*)) are the coordinates of the star spot at time *t_k_* in the image plane and *f* is the MEISS’s focal length. 

Let **A**(*t_k_*) represent the MEISS’s attitude matrix at time *t_k_*, and then the relation between **W_i_**(*t_k_*) and **V_i_** can be expressed as:

(3) $$W_{i}(t_{k})=A(t_{k})V_{i}\;(k=1,2,\ldots ,N;\;i=1,2,\ldots ,M),$$

where *M* represents the total number of guide stars in the MEISS’s field of view (FOV). 

Similarly, when the imaging time turns out to be *t_k_*_+1_, the relation between **W_i_**(*t_k_*_+1_) and **V_i_** can also be expressed as:

(4) $$W_{i}(t_{k+1})=A(t_{k+1})V_{i},$$

where **A**(*t_k_*_+1_) represents the MEISS’s attitude matrix at time *t_k_*_+1_. 

When substituting Equation (3) into Equation (4), it can be derived as:

(5) $$W_{i}(t_{k+1})=A(t_{k+1})V_{i}=A(t_{k+1})A{\left(t_{k}\right)}^{T}W_{i}(t_{k}).$$

If defining **R**(*t_k_*,*t_k_*_+1_) as the attitude rotation matrix of the star sensor from time *t_k_* to time *t_k_*_+1_, it can be expressed as:

(6) $$R(t_{k},t_{k+1})=A(t_{k+1})A{\left(t_{k}\right)}^{T}.$$

Since the time interval between time *t_k_* and time *t_k_*_+1_ is quite small, the rotation matrix can thus satisfy [43]:

(7) $$R(t_{k},t_{k+1})=Ι+⟦\Delta \theta ⟧+O({\left|\Delta \theta \right|}^{2}),$$

where **I** is the 3 × 3 identity matrix, Δ**θ** = [Δ*θ_x_* Δ*θ_y_* Δ*θ_z_*]^T^ is the small rotation angle vector, [[Δ**θ**]] is the antisymmetric matrix of Δ**θ**, and $O({\left|\Delta \theta \right|}^{2})$ is the higher-order infinitesimal matrix of Δ**θ**. 

Within the quite small time interval Δ*t* = *t_k_*_+1_ − *t_k_*, the motion of the star sensor can be considered as the uniform angular acceleration motion. Therefore, let **ω**(*t_k_*) = [*ω_x_*(*t_k_*) *ω_y_*(*t_k_*) *ω_z_*(*t_k_*)]^T^ and **α**(*t_k_*) = [*α_x_*(*t_k_*) *α_y_*(*t_k_*) *α_z_*(*t_k_*)]^T^ represent the angle velocity and angle acceleration of the star sensor at time *t_k_*, respectively, and then the instantaneous angle velocity **ω**(*t*) = [*ω_x_*(*t*) *ω_y_*(*t*) *ω_z_*(*t*)]^T^ can be expressed as:

(8) $$\omega (t)=\omega (t_{k})+\alpha (t_{k})(t-t_{k})\;(t_{k}\leq t\leq t_{k+1}).$$

By integrating Equation (8), the small rotation angle vector can be derived as:

(9) $$\Delta \theta =\int_{t_{k}}^{t_{k+1}}\omega (t)dt=\int_{t_{k}}^{t_{k+1}}(\omega (t_{k})+\alpha (t_{k})(t-t_{k}))dt=\omega (t_{k})\Delta t+\frac{1}{2}\alpha (t_{k})\Delta t^{2}\;(\Delta t=t_{k+1}-t_{k}).$$

When ignoring the higher-order infinitesimal matrix $O({\left|\Delta \theta \right|}^{2})$, the rotation matrix **R**(*t_k_*,*t_k_*_+1_) can be accordingly approximated as:

(10) $$\begin{array}{l} R(t_{k},t_{k+1})=Ι+⟦\Delta \theta ⟧=\left[\begin{array}{ccc} 1 & \Delta \theta_{z} & -\Delta \theta_{y}\\ -\Delta \theta_{z} & 1 & \Delta \theta_{x}\\ \Delta \theta_{y} & -\Delta \theta_{x} & 1 \end{array}\right]\\ =\left[\begin{array}{ccc} 1 & \omega_{z}(t_{k})\Delta t+\frac{1}{2}\alpha_{z}(t_{k})\Delta t^{2} & -\left[\omega_{y}(t_{k})\Delta t+\frac{1}{2}\alpha_{y}(t_{k})\Delta t^{2}\right]\\ -\left[\omega_{z}(t_{k})\Delta t+\frac{1}{2}\alpha_{z}(t_{k})\Delta t^{2}\right] & 1 & \omega_{x}(t_{k})\Delta t+\frac{1}{2}\alpha_{x}(t_{k})\Delta t^{2}\\ \omega_{y}(t_{k})\Delta t+\frac{1}{2}\alpha_{y}(t_{k})\Delta t^{2} & -\left[\omega_{x}(t_{k})\Delta t+\frac{1}{2}\alpha_{x}(t_{k})\Delta t^{2}\right] & 1 \end{array}\right]. \end{array}$$

When substituting Equations (2) and (10) into Equation (5), it can be derived as:

(11) $$\frac{1}{\sqrt{x_{i}{\left(t_{k+1}\right)}^{2}+y_{i}{\left(t_{k+1}\right)}^{2}+f^{2}}}\left[\begin{array}{c} -x_{i}(t_{k+1})\\ -y_{i}(t_{k+1})\\ f \end{array}\right]=\frac{1}{\sqrt{x_{i}{\left(t_{k}\right)}^{2}+y_{i}{\left(t_{k}\right)}^{2}+f^{2}}}\left[\begin{array}{ccc} 1 & \Delta \theta_{z} & -\Delta \theta_{y}\\ -\Delta \theta_{z} & 1 & \Delta \theta_{x}\\ \Delta \theta_{y} & -\Delta \theta_{x} & 1 \end{array}\right]\left[\begin{array}{c} -x_{i}(t_{k})\\ -y_{i}(t_{k})\\ f \end{array}\right].$$

According to Equation (11), the relationship of the third component of the observation unit vector can be expressed as:

(12) $$\frac{f}{\sqrt{x_{i}{\left(t_{k+1}\right)}^{2}+y_{i}{\left(t_{k+1}\right)}^{2}+f^{2}}}=\frac{1}{\sqrt{x_{i}{\left(t_{k}\right)}^{2}+y_{i}{\left(t_{k}\right)}^{2}+f^{2}}}\left(-\Delta \theta_{y}x_{i}(t_{k})+\Delta \theta_{x}y_{i}(t_{k})+f\right).$$

Furthermore, Equation (12) can also be derived as:

(13) $$\frac{\sqrt{x_{i}{\left(t_{k+1}\right)}^{2}+y_{i}{\left(t_{k+1}\right)}^{2}+f^{2}}}{\sqrt{x_{i}{\left(t_{k}\right)}^{2}+y_{i}{\left(t_{k}\right)}^{2}+f^{2}}}=\frac{f}{-\Delta \theta_{y}x_{i}(t_{k})+\Delta \theta_{x}y_{i}(t_{k})+f}=\frac{1}{\frac{-\Delta \theta_{y}x_{i}(t_{k})+\Delta \theta_{x}y_{i}(t_{k})}{f}+1}.$$

When substituting Equation (13) into Equation (11), it can be transformed as:

(14) $$\left[\begin{array}{c} -x_{i}(t_{k+1})\\ -y_{i}(t_{k+1})\\ f \end{array}\right]=\frac{1}{\frac{-\Delta \theta_{y}x_{i}(t_{k})+\Delta \theta_{x}y_{i}(t_{k})}{f}+1}\left[\begin{array}{ccc} 1 & \Delta \theta_{z} & -\Delta \theta_{y}\\ -\Delta \theta_{z} & 1 & \Delta \theta_{x}\\ \Delta \theta_{y} & -\Delta \theta_{x} & 1 \end{array}\right]\left[\begin{array}{c} -x_{i}(t_{k})\\ -y_{i}(t_{k})\\ f \end{array}\right].$$

Since the rotation angles Δ*θ_x_* and Δ*θ_y_* are quite small, the maximum calculation result of $\frac{-\Delta \theta_{y}x_{i}(t_{k})+\Delta \theta_{x}y_{i}(t_{k})}{f}$ is still less than 0.001 and can thus be ignored. Therefore, Equation (14) can be accordingly approximated as:

(15) $$\left[\begin{array}{c} -x_{i}(t_{k+1})\\ -y_{i}(t_{k+1})\\ f \end{array}\right]=\left[\begin{array}{ccc} 1 & \Delta \theta_{z} & -\Delta \theta_{y}\\ -\Delta \theta_{z} & 1 & \Delta \theta_{x}\\ \Delta \theta_{y} & -\Delta \theta_{x} & 1 \end{array}\right]\left[\begin{array}{c} -x_{i}(t_{k})\\ -y_{i}(t_{k})\\ f \end{array}\right].$$

Furthermore, considering that the focal length *f* is constant and (*x_i_*(*t*),*y_i_*(*t*)) are the coordinates of the star spot in the image plane, Equation (15) can be accordingly rewritten as:

(16) $$\left[\begin{array}{c} -x_{i}(t_{k+1})\\ -y_{i}(t_{k+1}) \end{array}\right]=\left[\begin{array}{ccc} 1 & \Delta \theta_{z} & -\Delta \theta_{y}\\ -\Delta \theta_{z} & 1 & \Delta \theta_{x} \end{array}\right]\left[\begin{array}{c} -x_{i}(t_{k})\\ -y_{i}(t_{k})\\ f \end{array}\right].$$

For clarity, Equation (16) can also be simplified as a two-dimensional formula:

(17) $$\left[\begin{array}{c} x_{i}(t_{k+1})\\ y_{i}(t_{k+1}) \end{array}\right]=\left[\begin{array}{cc} 1 & \Delta \theta_{z}\\ -\Delta \theta_{z} & 1 \end{array}\right]\left[\begin{array}{c} x_{i}(t_{k})\\ y_{i}(t_{k}) \end{array}\right]+\left[\begin{array}{cc} f & 0\\ 0 & -f \end{array}\right]\left[\begin{array}{c} \Delta \theta_{y}\\ \Delta \theta_{x} \end{array}\right].$$

Therefore, when the current coordinates (*x_i_*(*t_k_*),*y_i_*(*t_k_*)), the focal length *f*, and the small rotation angle vector Δ**θ** are known, the next coordinates (*x_i_*(*t_k_*_+1_),*y_i_*(*t_k_*_+1_)) can be predicted according to Equation (17). 

It should be noticed that the star point of the MEISS is generally 7 × 7 pixels in size. In order to fully contain the effective pixel data of the star point, each star point prediction window can thus be set at a size of 14 × 14 pixels, which is two times bigger than the size of the star point. 

### 2.2. Centroiding 

After the star point prediction windows are generated according to Equation (17), they can be used to process the next star image. Only pixel data within each prediction window should be used to calculate the real star centroid, while those pixel data without the prediction window must belong to fake star points and should thus be discarded. 

When obtaining the effective pixel data of the star spot, the classic centroid method [35] can be used to calculate the star centroid, which is expressed as:

(18) $$\{\begin{array}{c} x_{ic}(t_{k+1})=\frac{\sum m\cdot f(m,n)}{\sum f(m,n)}\\ y_{ic}(t_{k+1})=\frac{\sum n\cdot f(m,n)}{\sum f(m,n)} \end{array},$$

where *m*, *n*, and *f*(*m*, *n*) are the *x*-coordinate, *y*-coordinate, and grayscale value of each effective pixel in the prediction window, and (*x_i__c_*(*t_k_*_+1_),*y_i__c_*(*t_k_*_+1_)) are the calculated star centroid. Since the above centroid results inevitably include many image noises, they are just coarse centroids.

### 2.3. Kalman Filter

In order to improve the accuracy of the above coarse centroids, Kalman Filter is utilized to filter the image noises and thus obtain the fine centroids. Kalman Filter consists of a state equation and a measurement equation. 

Firstly, Equation (17) describes the relationship between the current centroid and the next centroid, and can thus be used as the state equation. Therefore, the state equation can be expressed as:

(19) $$X_{k+1}=\Phi_{k+1,k}X_{k}+B_{k}U_{k}+W_{k},$$

where **X***_k_*_+1_ = [*x_i_*(*t_k_*_+1_), *y_i_*(*t_k_*_+1_)]^T^ and **X***_k_* = [*x_i_*(*t_k_*), *y_i_*(*t_k_*)]^T^ are the state vectors at time *t_k_* and *t_k_*_+1_, respectively; **Φ***_k_*_+1,*k*_ is the state transfer matrix, which is expressed as:

(20) $$\Phi_{k+1,k}=\left[\begin{array}{cc} 1 & \Delta \theta_{z}\\ -\Delta \theta_{z} & 1 \end{array}\right]=\left[\begin{array}{cc} 1 & \omega_{z}(t_{k})\Delta t+\frac{1}{2}\alpha_{z}(t_{k})\Delta t^{2}\\ -\left[\omega_{z}(t_{k})\Delta t+\frac{1}{2}\alpha_{z}(t_{k})\Delta t^{2}\right] & 1 \end{array}\right];$$

**B***_k_***U***_k_* is the deterministic driving item, which is expressed as:

(21) $$B_{k}U_{k}=\left[\begin{array}{cc} f & 0\\ 0 & -f \end{array}\right]\left[\begin{array}{c} \Delta \theta_{y}\\ \Delta \theta_{x} \end{array}\right]=\left[\begin{array}{cc} f & 0\\ 0 & -f \end{array}\right]\left[\begin{array}{c} \omega_{y}(t_{k})\Delta t+\frac{1}{2}\alpha_{y}(t_{k})\Delta t^{2}\\ \omega_{x}(t_{k})\Delta t+\frac{1}{2}\alpha_{x}(t_{k})\Delta t^{2} \end{array}\right];$$

**W***_k_* represents the process noise, and its covariance matrix is **Q***_k_* = *ε***I**_2×2_, where **I**_2×2_ is a 2 × 2 identity matrix and *ε* is a very small positive quantity. 

Secondly, Equation (18) describes the measurement vector at time *t_k_*_+1_, and thus the measurement equation can be expressed as:

(22) $$Z_{k+1}=H_{k+1}X_{k+1}+V_{k+1},$$

where **Z***_k_*_+1_ = [*x_i__c_*(*t_k_*_+1_), *y_i__c_*(*t_k_*_+1_)]^T^ and **X***_k_*_+1_ = [*x_i_*(*t_k_*_+1_), *y_i_*(*t_k_*_+1_)]^T^ are the measurement vector and the state vector at time *t_k_*_+1_, respectively; **H***_k_*_+1_ = **I**_2×2_ is the measurement matrix; **V***_k_*_+1_ represents the measurement noise, and its covariance matrix is **R***_k_*_+1_ = *μ***I**_2×2_ (*μ* > 0), where *μ* is related to the noise level of the star image.

In summary, Equations (19) and (22) constitute the Kalman-Filter-based estimation model of the star centroid. The entire estimation process can be expressed as:

(23) $$\{\begin{array}{l} \hat{X}{\left(k+1\right)}_{k}=\Phi_{k+1,k}\hat{X}(k)+B_{k}U_{k}\\ P{\left(k+1\right)}_{k}=\Phi_{k+1,k}P(k)\Phi_{k+1,k}^{T}+Q_{k}\\ K(k+1)=P{\left(k+1\right)}_{k}{\left(P{\left(k+1\right)}_{k}+R_{k+1}\right)}^{-1}\\ P(k+1)=\left(I-K(k+1)\right)P{\left(k+1\right)}_{k}\\ \hat{X}(k+1)=\hat{X}{\left(k+1\right)}_{k}+K(k+1)\left(Z_{k+1}-\hat{X}{\left(k+1\right)}_{k}\right) \end{array},$$

where *k* and *k* + 1 represent the current and next exposure times, respectively; $\hat{X}(k)$ and $\hat{X}(k+1)$ are the state vector estimations, which correspond to the two exposure times *k* and *k* + 1, respectively; $\hat{X}{\left(k+1\right)}_{k}$ and $P{\left(k+1\right)}_{k}$ are the one-step prediction vector and one-step predictive error matrix, respectively; and P(k+1) and K(k+1) are the estimated error matrix and the filter gain matrix, respectively. When the initial values $\hat{X}(0)$ and P(0) are given, the star centroid vector $\hat{X}(k+1)$ can be estimated according to Equation (23). 

## 3. Simulations and Experiments

### 3.1. Simulations

In this section, simulations are conducted to verify the feasibility and effectiveness of the above Kalman-Filter-based estimation model. The simulated parameters of the MEISS are listed in Table 1. The MEISS’s total period *T* and the multi-exposure times *N* are set as 100 ms and 10, respectively, and thus the sampling interval between each exposure time is 10 ms. 

The entire simulations are run on a computer with a Windows 10 operating system and a CPU with a main frequency of 2.60 GHz. MATLAB (version: R2014a) is chosen as the simulation calculation software. The simulation process can be divided into several steps. Firstly, based on the star catalog of the Smithsonian Astrophysical Observatory (SAO), only stars with a magnitude of less than 6.0 are selected to build the MEISS’s navigation star catalog. Then, a set of attitude data is randomly generated as the MEISS’s initial attitude data, and a total of *M* = 30 navigation stars within the FOV are obtained under that attitude. Thirdly, the initial values of the angular velocity **ω**(*t*_0_) and angular acceleration **α**(*t*_0_) are assumed to be [10°/s 0 0]^T^ and [1.0°/s^2^ 0 0]^T^, respectively. Subsequently, among these 30 navigation stars, the 10th navigation star is randomly selected as the tested object. When the MEISS moves under the assumption of the above angular velocity **ω**(*t*_0_) and the angular acceleration **α**(*t*_0_), the centroid data of the 10th navigation star at continuous sampling times *S_t_*, whose total sampling times are set as 300, can be obtained and used as the true values. Lastly, three kinds of different sizes of Gaussian white noise are added to the true centroid data and the Kalman-Filter-based estimation model is used to filter the noisy data. 

**Figure 3:**
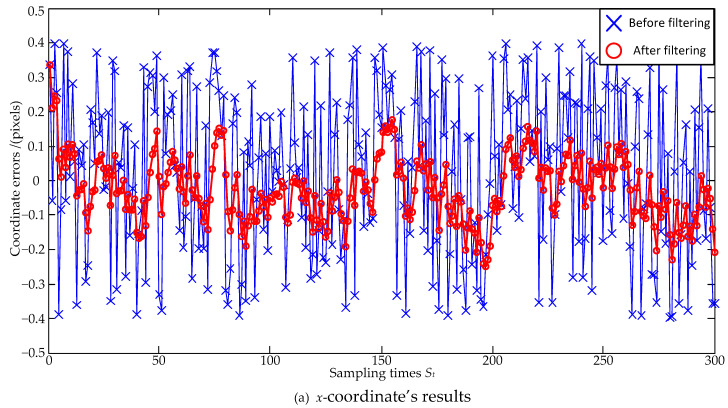

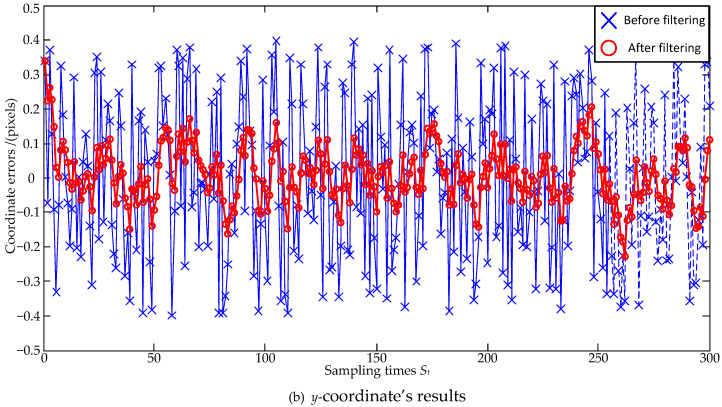
Coordinate estimation errors under ±0.4 pixel maximum deviation.

**Figure 4:**
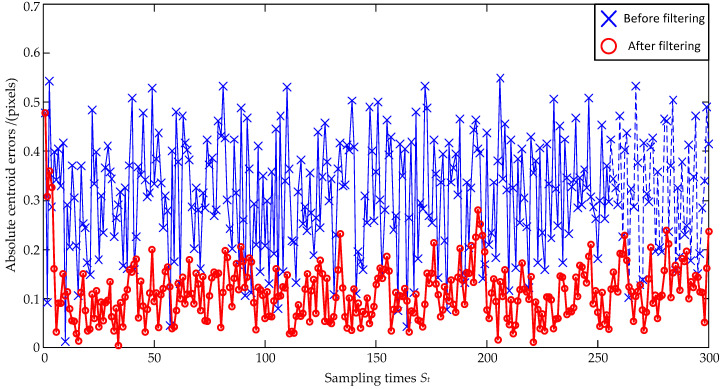
Absolute centroid errors under ±0.4 pixel maximum deviation.

The noise size is firstly set as a zero mean and ±0.4 pixel maximum deviation, and the estimation errors are shown in Figure 3 and Figure 4. In Figure 3, the standard deviations of the *x*-coordinate’s estimation errors are 0.24 pixels before filtering and 0.09 pixels after filtering, respectively; the standard deviations of the *y*-coordinate’s estimation errors are 0.23 pixels before filtering and 0.08 pixels after filtering, respectively. Both the standard deviations of the *x*-coordinate’s and the *y*-coordinate’s estimation errors are decreased, and the *x*-coordinate’s and the *y*-coordinate’s reductions can be, respectively, calculated as:

(24) $$\frac{0.09}{0.24}\times 100\%=37.5\%,\;\frac{0.08}{0.23}\times 100\%=34.8\%.$$

In order to make a reasonable evaluation, the larger value of 37.5% is selected as the evaluation result. That is to say, the coordinate errors are reduced to 37.5% of the original ones after the Kalman Filter estimation. In Figure 4, the absolute distance errors between the true and real values of the centroid are calculated, and it can be seen that the absolute distance errors are significantly reduced after filtering. 

**Figure 5:**
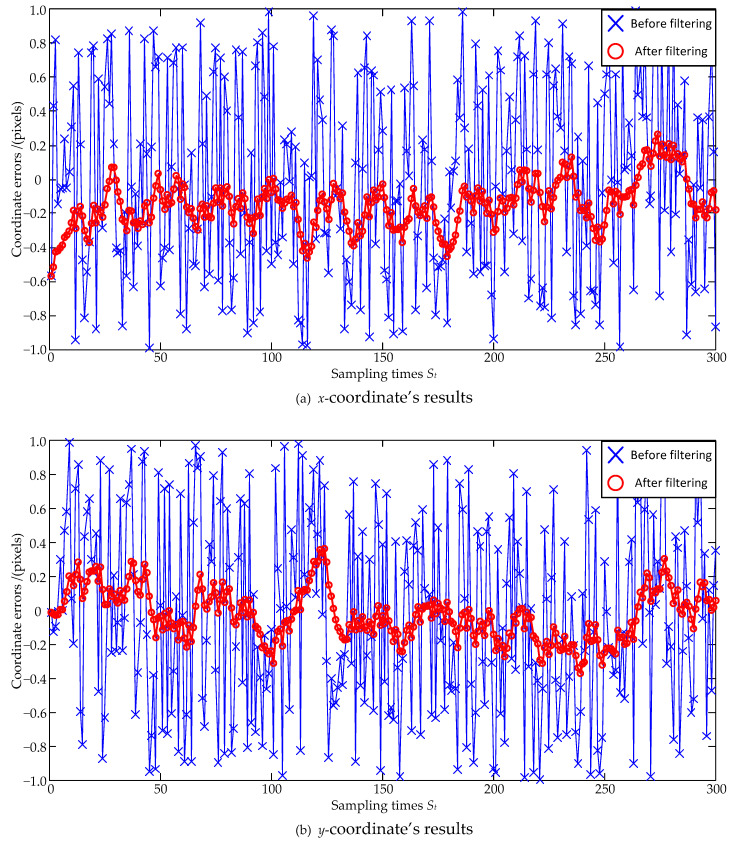
Coordinate estimation errors under ±1.0 pixel maximum deviation.

**Figure 6:**
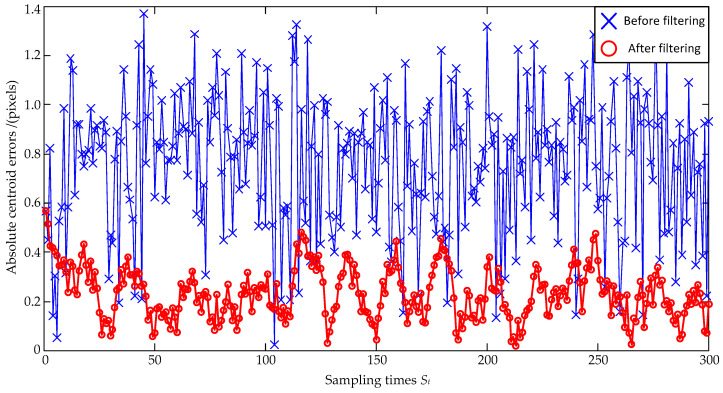
Absolute centroid errors under ±1.0 pixel maximum deviation.

Subsequently, the noise size is enlarged as a zero mean and ±1.0 pixel maximum deviation, and the estimation errors are shown in Figure 5 and Figure 6. In Figure 5, the standard deviations of the *x*-coordinate’s estimation errors are 0.57 pixels before filtering and 0.14 pixels after filtering, respectively; the standard deviations of the *y*-coordinate’s estimation errors are 0.57 pixels before filtering and 0.15 pixels after filtering, respectively. Both the standard deviations of the *x*-coordinate’s and the *y*-coordinate’s estimation errors are decreased, and the *x*-coordinate’s and the *y*-coordinate’s reductions can be, respectively, calculated as:

(25) $$\frac{0.14}{0.57}\times 100\%=24.6\%,\;\frac{0.15}{0.57}\times 100\%=26.3\%.$$

Similarly, the larger value of 26.3% is selected as the evaluation result. Therefore, the coordinate errors are reduced to 26.3% of the original ones after the Kalman Filter estimation. In Figure 6, the absolute distance errors are also significantly reduced after filtering. 

**Figure 7:**
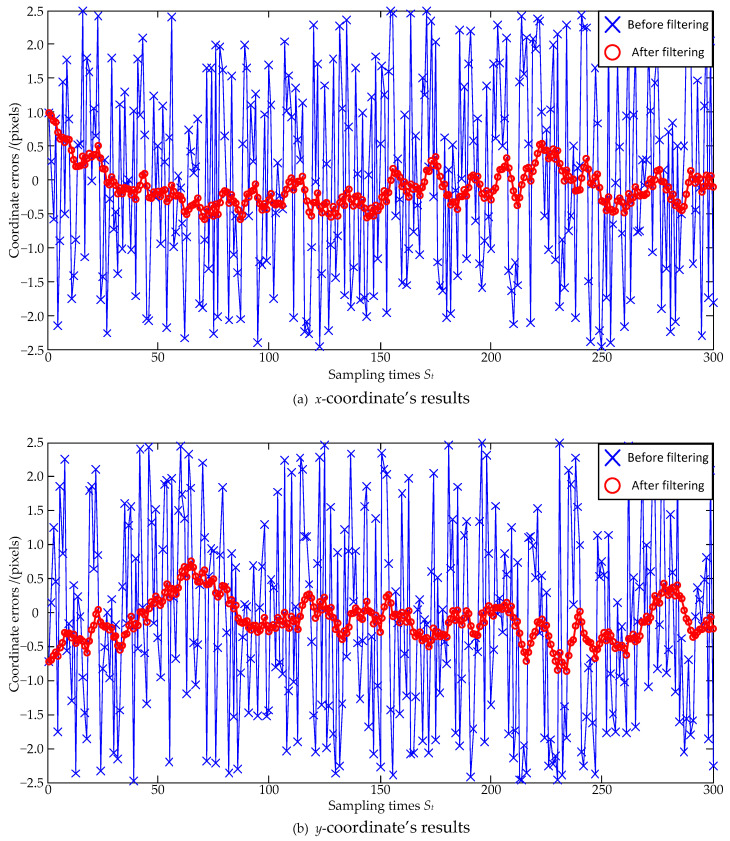
Coordinate estimation errors under ±2.5 pixel maximum deviation.

**Figure 8:**
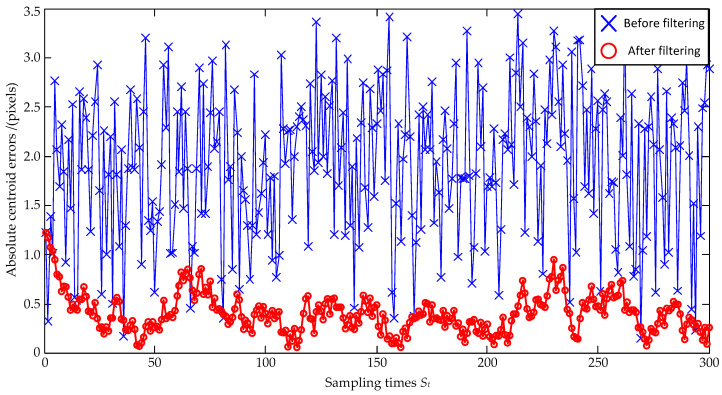
Absolute centroid errors under ±2.5 pixel maximum deviation.

Lastly, to simulate the performance of the Kalman-Filter-based estimation model under very large errors, the noise size is further enlarged as a zero mean and ±2.5 pixel maximum deviation, and the estimation errors are shown in Figure 7 and Figure 8. In Figure 7, the standard deviations of the *x*-coordinate’s estimation errors are 1.46 pixels before filtering and 0.29 pixels after filtering, respectively; the standard deviations of the *y*-coordinate’s estimation errors are 1.45 pixels before filtering and 0.30 pixels after filtering, respectively. Both the standard deviations of the *x*-coordinate’s and the *y*-coordinate’s estimation errors are decreased, and the *x*-coordinate’s and the *y*-coordinate’s reductions can be, respectively, calculated as:

(26) $$\frac{0.29}{1.46}\times 100\%=19.9\%,\;\frac{0.30}{1.45}\times 100\%=20.7\%.$$

Similarly, the coordinate errors are reduced to 20.7% of the original ones after the Kalman Filter estimation. In Figure 8, the absolute distance errors are also significantly reduced after filtering. 

According to the results of Figure 4, Figure 6, and Figure 8, the mean values and standard deviations of the absolute distance errors under different size noises are listed in Table 2. As shown in Table 2, the absolute distance errors of the Kalman Filter estimation increase with an increase in noises, but the increase in amplitude is smaller than that in the noises. When the noise size is enlarged as a zero mean and ±2.5 pixel maximum deviation, the mean values are 1.92 pixels before filtering and 0.40 pixels after filtering, respectively, and the standard deviations are 0.76 pixels before filtering and 0.20 pixels after filtering, respectively. It can be seen that the mean values and standard deviations of the absolute distance errors are significantly reduced after the Kalman Filter estimation. 

### 3.2. Experiments

**Figure 9:**
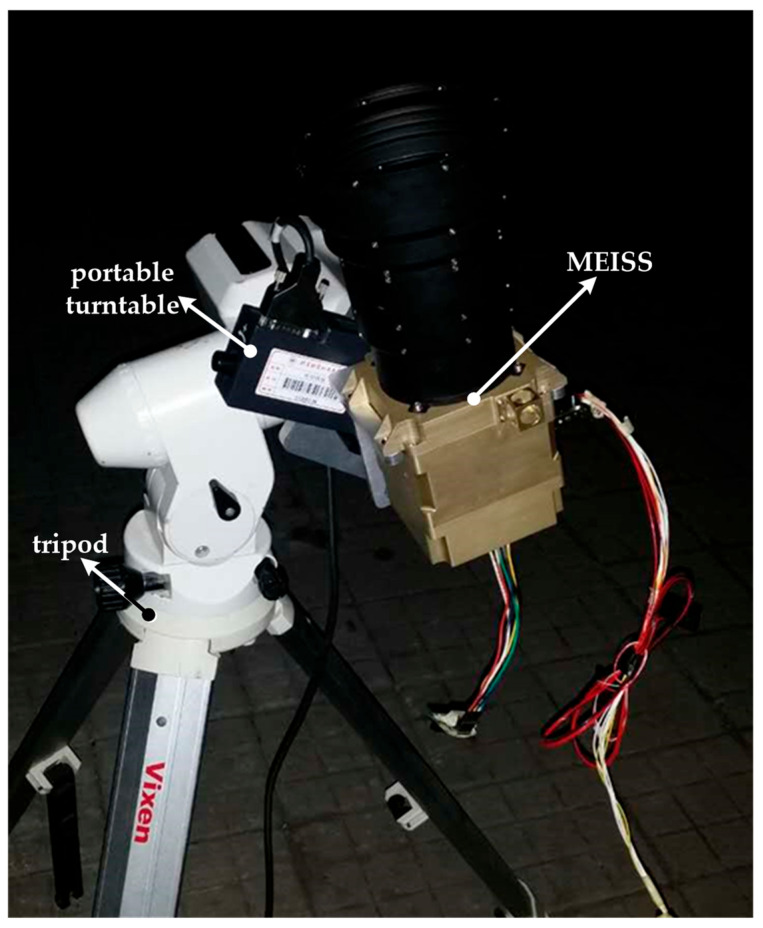
Night sky experiments.

In this section, experiments are conducted to further verify the feasibility and effectiveness of the star point prediction windows. Figure 9 shows the equipment of night sky experiments. The main experimental equipment includes the MEISS, a portable turntable, a tripod, a computer, and a power supply. The main design parameters adopted by the real MEISS in Figure 9, such as the focal length and the FOV, etc., are the same as those listed in Table 1. A portable turntable is firmly installed on a tripod. The MEISS is installed on the turntable and rotates with it. The computer is the same as the one used in the simulations. The power supply is used to provide 220 V AC and 5 V DC. 

**Figure 10:**
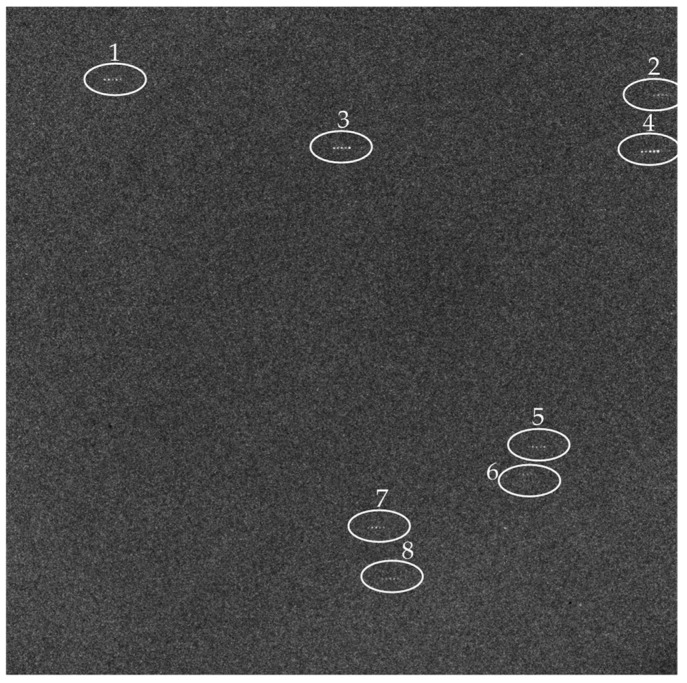
One multi-exposure star image.

The angular velocity and angular acceleration of the turntable are set as 5°/s and 0.5°/s^2^, respectively, and the multi-exposure times *N* is set as 5. Under these conditions, the MEISS begins to work, and one of the star images obtained by MEISS is shown as Figure 10. 

In Figure 10, there are, in total, eight guide stars within the star image, which are numbered as 1–8, and the rest is a starry background with noise. Since the multi-exposure times *N* is set as five, each guide star has five star spots. Furthermore, it can be seen that the noise level of the star image is relatively high. When using the global threshold segmentation (global threshold = 128) and classic centroid method, there are a total of 4985 objects. Among them, there are 3664 one-pixel objects accounting for 73.5% of the total, 749 two-pixel objects accounting for 15.0% of the total, and 572 over-three-pixel objects accounting for 11.5% of the total. Although most objects are false (one-pixel and two-pixel objects) and can be eliminated, the number of candidate star point objects is still large (572 over-three-pixel objects), which seriously hinders the accurate extraction of real star point objects. In contrast, when using the star point prediction windows for centroid extraction, only 8 × 5 = 40 star point objects are extracted, accounting for only 0.8% of the total. Similarly, under the same experimental conditions, another 99 star images are obtained to further verify the star point prediction windows. Among the 100 star images, the average proportion of the number of effective star point objects in the total object number of each star image is calculated as only 0.95%, which greatly demonstrates the effectiveness of the proposed method. 

## 4. Discussion

In the simulations, different size noises were added to the true centroid data to verify the performance of the Kalman-Filter-based estimation model. Under the noises with z zero mean and ±0.4, ±1.0, and ±2.5 pixel maximum deviations, the coordinate errors after filtering were reduced to about 37.5%, 26.3%, and 20.7% of the original ones, respectively. The absolute distance errors of the Kalman Filter estimation increased with an increase in noises, but the increase amplitude was smaller than that of noises. Under the noise with a zero mean and ±2.5 pixel maximum deviation, the mean value and standard deviation of the absolute distance errors before filtering were 1.92 pixels and 0.76 pixels, respectively, and the errors were too large to perform the correct star image recognition. In contrast, the mean value and standard deviation after filtering were 0.40 pixels and 0.20 pixels, respectively, which still remain at the sub pixel level of accuracy and thus enabled the correct star image recognition. 

Subsequently, night sky experiments were conducted to verify the performance of the star point prediction windows. Taking one MEISS’s star image as an example, its noise level was relatively high. When the classic centroid method was used, a total 4985 objects were obtained. Among them, the number of candidate star point objects was 572 (accounting for 11.5% of the total), which seriously hindered the accurate extraction of real star point objects. Furthermore, due to the high level of noise, the global threshold had to be set as 128, and this would filter out the weak pixel signals of the real star points, resulting in a decrease in the centroid accuracy. In contrast, when using the star point prediction windows for the centroid extraction, the local threshold could be adaptively set within a very small prediction window, and only 8 × 5 = 40 star point objects were extracted, accounting for only 0.8% of the total. Similarly, under the same experimental conditions, a total of 100 star images were obtained, and the average proportion of the number of effective star point objects in the total object number of each star image was calculated as only 0.95%. 

Both the simulations and experiments verified the feasibility and effectiveness of the proposed method. It should be noticed that the proposed method also had certain limitations. In order to effectively filter out the noise carried by the centroid data, it was necessary to perform the filtering process on each star point object, thus resulting in significant computational and time costs. Therefore, further research work should focus on parallel computing based on Field Programmable Gate Array (FPGA) and improving the computational efficiency of the Kalman-Filter-based estimation model. 

## 5. Conclusions

A multi-exposure imaging approach, as proposed in earlier studies, can be used to increase the AUR by *N* times. However, serious noises are also introduced into star images due to multiple exposures. In this paper, a high-accuracy star centroid extraction method based on Kalman Filter was proposed to remove false objects and improve the centroid accuracy, which is beneficial to the MEISS’s practical application. The principle of the proposed method was described in detail, including star point prediction windows, centroiding, and Kalman Filter. Subsequently, simulations were conducted to verify the performance of the Kalman-Filter-based estimation model. Furthermore, experiments were also conducted to verify the performance of the star point prediction windows. Both the simulated and experimental results demonstrated the feasibility and effectiveness of the proposed method. 

## Figures and Tables

**Table 1:** MEISS’s simulated parameters.

Parameter	Value
Period *T* (ms)	100
Multi-exposure times *N*	10
Resolution *S* × *S* (pixels)	2048 × 2048
Pixel size *a* × *a* (μm)	5.5 × 5.5
Focal length *f* (mm)	31.94
FOV (°)	20

**Table 2:** Mean values and standard deviations of absolute distance errors.

Item	±0.4 Pixel Noise		±1.0 Pixel Noise		±2.5 Pixel Noise	
	Before Filtering	After Filtering	Before Filtering	After Filtering	Before Filtering	After Filtering
Mean value (pixels)	0.31	0.11	0.76	0.23	1.92	0.40
Standard deviation (pixels)	0.11	0.06	0.28	0.10	0.76	0.20

## Data Availability

Not applicable.
